# Use of Glucarpidase for Methotrexate-Induced Nephrotoxicity During the Treatment of Primary Central Nervous System Lymphoma

**DOI:** 10.7759/cureus.100718

**Published:** 2026-01-03

**Authors:** Yusuke Fujiwara, Akane Takaono, Akimitsu Kitagawa, Muneo Okamoto

**Affiliations:** 1 Department of Hematology, Nippon Medical School, Tokyo, JPN; 2 Department of Hematology, Shonantobu General Hospital, Chigasaki, JPN; 3 Department of Internal Medicine, Shonantobu General Hospital, Chigasaki, JPN; 4 Department of Nephrology, Shonantobu General Hospital, Chigasaki, JPN

**Keywords:** drug induced aki, glucarpidase, high dose methotrexate, methotrexate (mtx), pcnsl management, primary central nervous system lymphoma (pcnsl)

## Abstract

High-dose methotrexate (HD-MTX)-based combination chemotherapy remains the mainstay of treatment for primary central nervous system lymphoma (PCNSL). However, MTX-induced nephrotoxicity can occur even with adequate preventive and supportive measures. We report a case of MTX-induced acute kidney injury (AKI) that developed during treatment for PCNSL. During the second course of chemotherapy, serum creatinine increased from 0.74 mg/dL at baseline to 2.15 mg/dL on day 3, accompanied by a peak plasma MTX concentration of 3.89 μmol/L. Following administration of glucarpidase, plasma MTX levels declined to 0.08 μmol/L by day 12, and renal function subsequently improved, with serum creatinine decreasing to 1.47 mg/dL by day 19. This biochemical improvement allowed continuation of MTX-based chemotherapy, and the patient ultimately achieved complete remission after seven courses of treatment. This case highlights the role of glucarpidase in facilitating treatment continuity in the setting of MTX-induced nephrotoxicity and underscores the importance of prompt and appropriate supportive care in the management of PCNSL.

## Introduction

Primary central nervous system lymphoma (PCNSL) is a rare subtype of non-Hodgkin lymphoma confined to the central nervous system (CNS), with over 90% of cases histologically classified as diffuse large B-cell lymphoma (DLBCL). PCNSL accounts for approximately 4% of CNS tumors and 4-6% of extranodal lymphomas, and its incidence is increasing, particularly among the elderly in Japan [1,2].

High-dose methotrexate (HD-MTX)-based multi-agent chemotherapy remains the standard induction therapy for PCNSL. Among these regimens, R-MPV therapy, comprising rituximab, HD-MTX, procarbazine, and vincristine, has demonstrated favorable response rates and improved outcomes and is widely adopted internationally [3-5]. HD-MTX, a cytotoxic agent capable of penetrating the blood-brain barrier (BBB), serves as the cornerstone of this regimen. Following induction, consolidation therapy, including whole-brain radiotherapy (WBRT) or autologous hematopoietic stem cell transplantation (AHCT), is recommended to improve long-term survival [5-7].

However, HD-MTX carries a risk of significant toxicity, with acute kidney injury (AKI) being a major adverse effect that may hinder treatment continuity. Since MTX is primarily excreted via the kidneys, intratubular precipitation can lead to renal impairment. Once renal function is compromised, delayed MTX clearance may induce severe toxicities such as myelosuppression, gastrointestinal injury, hepatotoxicity, and neurotoxicity. Therefore, preventive strategies including aggressive hydration, urine alkalinization, leucovorin rescue, and therapeutic drug monitoring of MTX are essential. Nevertheless, approximately 10% of patients still develop AKI, occasionally requiring renal replacement therapy [8-13].

Recently, glucarpidase, a bacterial enzyme that hydrolyzes MTX, has emerged as an effective therapeutic option for HD-MTX-related nephrotoxicity. Glucarpidase is a recombinant Variovorax paradoxus glutamate carboxypeptidase that hydrolyzes the terminal glutamate residue of MTX, thereby enabling renal-independent metabolism and promoting rapid plasma clearance of MTX to prevent systemic toxicity [8,9,11,14].

Given the poor prognosis of PCNSL, completion of R-MPV therapy and timely initiation of consolidation are key factors for achieving optimal outcomes. Rapid and appropriate management of HD-MTX-associated toxicities is critical for maintaining treatment continuity and improving prognosis. Proper implementation of supportive therapies is, therefore, essential [3-6,8].

In the present case, the patient developed HD-MTX-induced AKI during R-MPV therapy, which did not improve with conventional supportive measures. Administration of glucarpidase led to improvement in renal function and reduction in MTX levels (i.e., MTX concentrations), allowing the continuation of all seven planned R-MPV courses and ultimately achieving complete remission. This case highlights the clinical value of glucarpidase and underscores the importance of supportive strategies in the treatment of PCNSL.

## Case presentation

**Figure 1:**
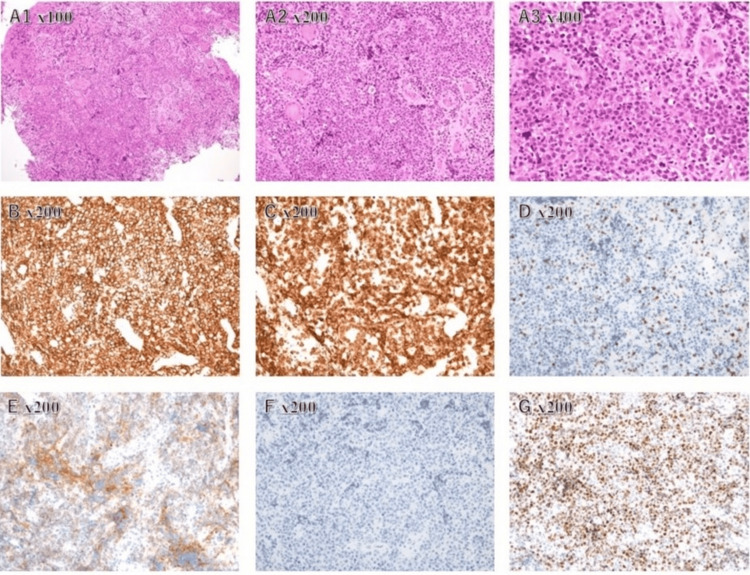
Histopathological findings of the brain tumor. (A1) ×100, (A2) ×200, (A3) ×400, hematoxylin and eosin staining; (B–G) ×200: (B) CD20, (C) CD79a, (D) CD5, (E) CD10, (F) BCL2, and (G) BCL6. The specimen demonstrated a dense proliferation of large atypical lymphoid cells with a high nuclear-to-cytoplasmic ratio. The tumor cells were positive for CD20 and CD79a, consistent with B-cell lineage, and negative for the T-cell marker CD5. Although BCL2 expression was absent, the cells were positive for CD10 and BCL6.

A 58-year-old man with no significant medical history developed a headache in August 2024. In September, he experienced gait disturbance with leftward leaning and frequent falls. As symptoms persisted, he visited a local hospital in December. Brain MRI revealed an enhancing mass lesion in the right basal ganglia, and a stereotactic brain biopsy was performed. Histopathology confirmed DLBCL, and a systemic workup revealed no extracranial involvement, leading to a diagnosis of PCNSL. The histopathological findings of the brain tumor are shown in Figure 1.

Initial treatment with dexamethasone (4 mg/day) was started at the referring hospital, but due to the absence of a department of hematology, the patient was transferred to our hospital on postoperative day 3. Upon admission, his left-sided hemiparesis had improved to grade 4 on manual muscle testing (MMT). Hydration and urine alkalinization were initiated, and R-MPV therapy (rituximab 375 mg/m², MTX 3,500 mg/m², vincristine 1.4 mg/m², and procarbazine 100 mg/m²) was administered. At baseline, prior to initiation of R-MPV therapy, the serum creatinine level was 0.93 mg/dL and the estimated glomerular filtration rate (eGFR) was 65.5 mL/min/1.73 m². After the first course, neurological symptoms improved, and tumor reduction was noted. MTX levels were not measured due to the year-end closure of external laboratories; however, clinical and radiological improvement supported continuation of treatment without major adverse events.

**Figure 2:**
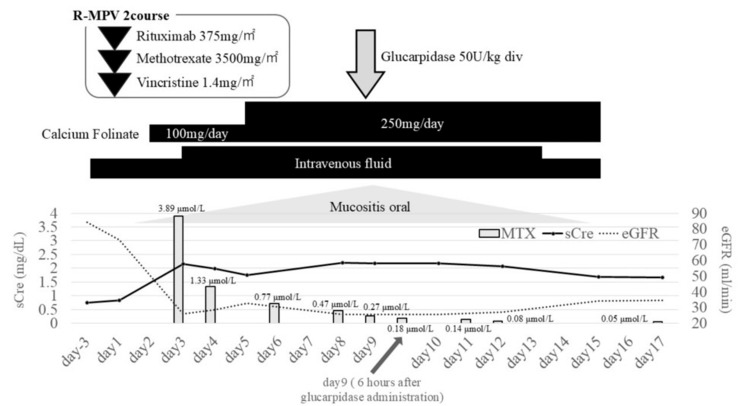
Clinical course during the second course of R-MPV therapy, from day −3 to temporary discharge. MTX: methotrexate; sCre: serum creatinine; eGFR: estimated glomerular filtration rate

In January 2025, the patient was readmitted for the second course. According to the R-MPV protocol, procarbazine is administered only during odd-numbered courses; therefore, it was omitted in the second course. Laboratory findings on admission are shown in Table 1, and Figure 2 shows the treatment course, including temporal changes in serum creatinine, eGFR, and MTX levels.

**Table 1:** Laboratory data on admission. eGFR: estimated glomerular filtration rate; sIL-2R: soluble interleukin-2 receptor

Parameter	Patient Value	Reference Range	
Complete blood count			
White blood cell	7710	3300-8600	/μL
Neutrophil	73.9	42-74	%
Lymphocyte	8.4	18-50	%
Monocyte	12.8	1-8	%
Eosinophil	3.1	0-7	%
Basophil	1.8	0-2	%
Red blood cells	361	435-555	×10⁴ /μL
Hemoglobin	11.3	13.7-16.8	g/dL
Platelet	41.8	15.8-34.8	×10⁴/μL
Biochemical tests			
Total protein	5.9	6.6-8.1	g/dL
Albumin	2.9	4.1-5.1	g/dL
Aspartate aminotransferase	102	13-30	U/L
Alanine aminotransferase	146	10-42	U/L
Total bilirubin	0.3	0.4-1.5	mg/dL
Alkaline phosphatase	108	38-113	U/L
Creatine phosphokinase	33	59-248	U/L
Lactate dehydrogenase	283	124-222	U/L
Blood urea nitrogen	16.2	8.0-20.0	mg/dL
Creatinine	0.74	0.65-1.07	mg/dL
eGFR	84.1	> 90	mL/min/1.73㎡
Sodium	136	138-145	mmol/L
Potassium	3.8	3.6-4.8	mmol/L
Chloride	103	101-108	mmol/L
C-reactive protein	0.87	0.00-0.14	mg/dL
sIL-2R	963.0	156.6-474.5	U/mL

Hydration, urine alkalinization, and leucovorin rescue therapy were provided. On day 3 post-MTX, the serum creatinine level increased to 2.15 mg/dL, and eGFR had decreased by more than 50%, consistent with Kidney Disease: Improving Global Outcomes (KDIGO) stage 2 AKI. At our institution, the expected MTX level targets are <1.0 µmol/L at 48 hours and <0.1 µmol/L at 72 hours after MTX administration. The MTX level at 48 hours was 3.89 µmol/L, exceeding the institutional target and consistent with MTX-related AKI. Urine output was sufficient at 5,100 mL, and there were no changes in MTX dosing or nephrotoxic concomitant medications.

Supportive therapy included escalation of leucovorin to 250 mg/day, intravenous hydration of 4,000 mL/day, and oral fluid intake of 2,000 mL/day. Leucovorin rescue and hydration were intensified when AKI developed on day 3; however, despite these interventions, the MTX level remained high at 1.33 µmol/L on day 4. By day 8, the level had decreased to 0.47 µmol/L; however, the patient developed grade 2 mucositis and grade 4 neutropenia. Although intensified supportive care was expected to promote MTX clearance and renal recovery, the clinical response was insufficient, and achieving <0.1 µmol/L with supportive measures alone was considered unlikely in the setting of persistent MTX exposure and renal dysfunction. In addition, at our institution, MTX levels are measured by an external laboratory, and accurate results typically require three to five days to become available. Non-contrast abdominal computed tomography showed no evidence of postrenal obstruction. As these factors delayed confirmation of persistent MTX exposure and continued treatment was considered difficult, glucarpidase (50 U/kg; total 3,000 U) was administered on day 9. Leucovorin administration was withheld for two hours before and two hours after glucarpidase infusion. Six hours after administration, the MTX level declined to 0.18 µmol/L and further to 0.08 µmol/L by day 12. Mucositis resolved by day 15, and neutropenia recovered by day 20. Renal function gradually improved. Before the third course, serum creatinine was 1.36 mg/dL and eGFR 43.2 mL/min; before the fifth course, they had improved to 0.99 mg/dL and 61.2 mL/min, respectively.

**Figure 3:**
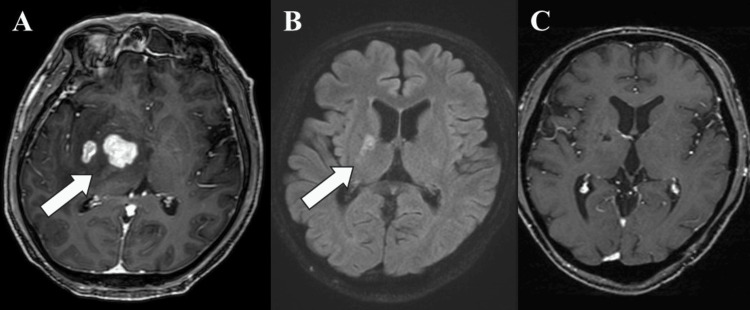
Serial contrast-enhanced T1-weighted magnetic resonance imaging (MRI) of the brain in the present case. Gadolinium-enhanced T1-weighted brain MRI at baseline (A), after five courses of R-MPV therapy (B), and after seven courses of R-MPV therapy (C). Arrows indicate the primary lesion.

From the third course onward, the treatment interval was unchanged, and the MTX dose was adjusted in each course according to renal function; treatment continued without further episodes of AKI or delayed MTX clearance. A partial response was achieved after five courses and a complete response after seven, as supported by serial MRI findings shown in Figure 3. After completion of seven courses, the pre-treatment left hemiparesis had resolved, with MMT graded as 5 (normal). Given renal recovery, consolidation therapy, including high-dose chemotherapy and AHCT, is planned at another institution.

## Discussion

This case involved a patient with PCNSL who developed AKI induced by HD-MTX during R-MPV therapy. Despite standard supportive care, including hydration and leucovorin rescue, renal function continued to deteriorate. The administration of glucarpidase was associated with an improvement in renal function and MTX clearance, enabling continuation of therapy. The patient ultimately completed seven courses of R-MPV and achieved complete remission.

R-MPV therapy is considered the international standard for induction treatment of PCNSL, with MTX as its core agent [3-5]. MTX is predominantly excreted by the kidneys; even minor renal impairment can result in drug accumulation, causing severe toxicities such as myelosuppression, mucositis, hepatotoxicity, and neurotoxicity. Although intensive hydration and urine alkalinization have substantially improved the safety of HD-MTX therapy, MTX-associated AKI still occurs in approximately 10% of patients despite appropriate preventive measures [8-10,12]. In some cases, renal replacement therapy becomes necessary, particularly when MTX clearance is markedly delayed. Importantly, the timing of onset is difficult to predict, and the occurrence of nephrotoxicity is not always reproducible across different treatment courses within the same patient. These factors may partly explain why delayed MTX clearance developed during the second course in the present case, despite the absence of significant toxicity during the first course.

Glucarpidase has recently emerged as a promising treatment option for MTX-related AKI. It is a recombinant bacterial enzyme (carboxypeptidase G2) that hydrolyzes MTX into inactive metabolites via a non-renal route, leading to rapid and profound reductions in MTX levels [8,9,11]. In this case, persistent elevation of MTX levels and progression of renal impairment prompted administration of glucarpidase, which resulted in effective MTX clearance and avoidance of irreversible AKI.

Recent studies have highlighted the potential utility of planned or reduced-dose glucarpidase administration. Scheduled low-dose glucarpidase (1,000-2,000 U) within 24 hours after MTX infusion has been shown to enhance drug clearance and facilitate continued therapy, even in outpatient settings [13-16]. This strategic use may reduce adverse events, shorten hospitalization, and conserve healthcare resources.

However, some limitations of glucarpidase use remain. The formation of anti-glucarpidase antibodies following repeated administration has been reported and may affect its efficacy [15]. Furthermore, as glucarpidase does not cross the BBB, it does not reduce MTX concentrations within the CNS. Therefore, it likely has limited direct impact on therapeutic efficacy for PCNSL [9].

This case is clinically significant in that glucarpidase administration was associated with a decline in MTX levels and subsequent improvement in renal function, allowing continuation of R-MPV therapy and ultimately achieving complete remission. This experience suggests that timely glucarpidase use, alongside supportive care, may help maintain treatment continuity when MTX-induced nephrotoxicity occurs during MTX-based chemotherapy for PCNSL.

## Conclusions

This case describes a patient with PCNSL who developed HD-MTX-induced AKI during R-MPV therapy. Glucarpidase administration was associated with a decline in MTX levels and subsequent recovery of renal function, allowing continuation of treatment and ultimately achieving complete remission. While HD-MTX-based chemotherapy remains the cornerstone of induction therapy for PCNSL, the risk of nephrotoxicity cannot be fully eliminated even with appropriate supportive care. Timely and appropriate management of HD-MTX-related adverse events is essential to maintain treatment continuity. Glucarpidase is a therapeutic option for MTX-induced renal toxicity and may help facilitate uninterrupted HD-MTX-based therapy in selected patients.
